# Supplementary material for: Research on health education and health promotion during the process of schistosomiasis elimination II Awareness among university students in endemic regions
Source: PLoS Negl Trop Dis. 2025 Feb 18;19(2):e0012865. doi: 10.1371/journal.pntd.0012865 (PMC11835237; doi:10.1371/journal.pntd.0012865)
Supplement: S1 Text — (PDF) [file pntd.0012865.s001.pdf]

A Questionnaire Survey on the Awareness of Schistosomiasis among College Students in Gongqingcheng City

Dear classmate:

Hello! We are conducting a survey on schistosomiasis awareness among university students in Gongqingcheng City, hoping to find out how much you know about schistosomiasis and your awareness of prevention. This will help us to better promote and prevent schistosomiasis and protect your health. Your participation will have a significant impact on our prevention and treatment efforts, and we thank you for your support!

Please take a few minutes to complete the following questionnaire, all information will be kept strictly confidential. Thank you very much!

By starting to complete this questionnaire, you are indicating that you understand your rights and responsibilities and that you agree to our survey.

Q1. Have you visited the surrounding areas of Poyang Lake, such as Nanhu, Sizhuang, Wanjia Lake, Sujiadang, etc., and done activities there? (Maximum 3 choices or select ″Not going″ alone) ((If you choose not going, the survey will end) [Multiple choice question] *

□The surrounding grasslands of Jiangyi Town

□Lake around Sujiadang

□Wucheng Town

□Sha Hu Shan

□Other Areas _________________*

□Not going

Q2. Your age group: [Single choice question] *

○Under 18 years old

○Ages 18-25

○26 years and above

Q3. Gender: [Single choice question] *

○Male

○Female

Q4. What is your current type of education? [Single choice question] *

○Bachelor's Degree / Undergraduate

○Postgraduate or above

Q5. Where did you come from to study at this school?

Province: _____________ Township (or town, field): _____________(If there is no specific township or town, please fill in "None") [Fill-in-the-blank] *

Q6. Which of the following activities have you done since arriving in the Poyang Lake District? (Maximum 3 choices or select) [Multiple choice question] *

☐Fishing and shrimping

☐Picking wild vegetables

☐Washing/handwashing

☐On-site practice

☐Flood control and waterlogging prevention

☐Swimming/water wading

☐Other (please specify:) _________________*

☐Just simply strolling, without the aforementioned activities

Q7. The main period you will be visiting the Poyang Lake area: [Single choice question] *

○A. January - March

○B. April - October

○C. November - December

○D. Year-round (irregular)

Q8. Have you ever heard of schistosomiasis? (If "No" is selected, the survey will be terminated) [Single choice question] *

○A. Yes

○B. No (Please proceed to the end of the questionnaire and submit your answers)

Q9. Do you know how people get schistosomiasis? [Single choice question] *

○A. Came into contact with the water of Poyang Lake

○B. Consumed freshwater fish and shrimp

○C. Ate wild vegetables

○D. Don't know

Q10. Are you aware that the area of Poyang Lake you are traveling to is a risk zone for schistosomiasis infection? [Single choice question] *

○A. Know

○B. Don't know

Q11. Which of the following organisms can spread schistosomiasis? [Single choice question] *

○A. Oncomelania hupensis

○B. Mosquito

○C. Leech

○D. Semisulcospira cancellata

○E. Don't know

Q12. What are the symptoms of schistosomiasis? [Single choice question] *

○A. Itchy skin, fever, abdominal pain, diarrhea

○B. Nausea and vomiting

○C. Dizziness and tinnitus

○D. Don't know

Q13. If you suspect that you have schistosomiasis, where would you generally go for a schistosomiasis test? [Single choice question] *

○A. Hospitals at or above the municipal level

○B. Community health stations (institutes)

○C. Disease control and prevention centers (malaria prevention stations) (schistosomiasis control station)

○D. Will not go

Q14. Have you ever seen a roadside sign or audio message about schistosomiasis control or received a leaflet about schistosomiasis control when entering this area? [Single choice question] *

○A. Yes

○B. No

Q15. How do you handle the issue of urination and defecation during outdoor activities? [Single choice question] *

○A. Urinate and defecate anywhere

○B. Find a nearby restroom

Q16. During your activities in the Poyang Lake area, do you take preventive measures against schistosomiasis? [Single choice question] *

○A. Yes

○B. No

Q17. Do you know what measures can be taken to prevent schistosomiasis when engaging in activities on the grasslands of Po Yang Lake area? [Multiple choice question] *

☐A. Do not contact contaminated water

☐B. Wear rubber gloves, rubber boots, and other protective equipment

☐C. Use preventive medication

☐D. Other (Please specify:) _________________*

☐E. Don't know

Q18. Which type of publicity to prevent schistosomiasis do you prefer? [Multiple choice question] *

☐A. Leaflet

☐B. Physical Protection Publicity Materials

☐C. Mobile Multimedia

☐D. Warning signs for schistosomiasis control erected in risk areas

☐E. Intelligent Schistosomiasis Control Voice Prompts Erected in Risk Areas
