# Supplementary material for: Research on health education and health promotion during the process of schistosomiasis elimination II Awareness among university students in endemic regions
Source: PLoS Negl Trop Dis. 2025 Feb 18;19(2):e0012865. doi: 10.1371/journal.pntd.0012865 (PMC11835237; doi:10.1371/journal.pntd.0012865)
Supplement: Attachment — Submitted filename: Review PNTD-D-24-01660.docx [file pntd.0012865.s003.docx]

**TOPIC**

Research on health education and health promotion during the process of schistosomiasis elimination II A study examining the awareness of schistosomiasis among students at higher education institutions in endemic regions.

The title is overly long and slightly ambiguous. Consider simplifying it while retaining key. Here is a suggestion:

***"Health Education and Promotion During Schistosomiasis Elimination: Awareness Among Students in Endemic Regions"*.**

**ABSTRACT**

The abstract provides a comprehensive overview of the research. However, it is rather too long with lots of redundancies. Authors are advised to **rephrase and summarize the abstract section.** There are areas where the clarity, and structure could be improved:

1) The objectives aspect introduces schistosomiasis and health education in China, but it lacks a clear framing of why university students are a critical focus for this study. **Briefly connecting this to their role as a potentially at-risk or influential group would enhance its relevance**.
2) The description of the questionnaire's content is helpful but could be more concise. For example, instead of listing all topics, summarize:
   *"The survey assessed exposure to snail-infested water, knowledge about schistosomiasis, and preferences for health education methods."*
3) Some statistical data (e.g., “χ²=0.620, P>0.05”) are included without sufficient context. If the finding is not significant, it might be better to emphasize the broader pattern rather than the statistical test result, which may distract a general audience. The result section can be summarized below:

*“Among 4,847 respondents (49% male, 51% female), 53.8% reported exposure to snail-infested environments near their schools, and 38.4% had direct contact with snail-infested water. Awareness of schistosomiasis was low (31.1%), with limited knowledge about transmission (30.6%) and symptoms (42.9%). Preferred education methods included mobile multimedia (72.8%), brochures (68.7%), and physical promotional items (66.6%)”.*

**INTRODUCTION**

The introduction effectively highlights schistosomiasis as a significant public health concern, contextualizing its global and regional impact. By referencing government initiatives and WHO strategies, it establishes credibility while emphasizing China's progress, including the 2023–2030 Action Programme for Schistosomiasis Elimination. The identified gaps in health education for mobile populations emphasize the study's importance and provide a clear rationale for its focus. However, there is need for some improvements:

1) While the gaps are identified, the justification for focusing specifically on university students could be stronger. Briefly linking this to their unique vulnerability or potential role as change agents in endemic areas would add value.
2) Paragraph 3: Kindly rephrase this sentence for more clarity "*China has gained much experience in health education and health promotion in the process of schistosomiasis prevention and treatment, which has provided some technical support for the elimination of schistosomiasis*"

**METHODS**

This section provides useful details about the participants, study design, tools, and ethical considerations. However, there are areas where clarity, precision, and completeness could be improved:

1) The choice of a non-probability sample survey is mentioned but not justified. Why this method was selected over a probability sampling method should be explained, as it affects the generalizability of the results.
2) While the questionnaire topics were briefly described, more detail is needed about how it was developed and validated. Also, the format and scope of the questions (e.g., multiple choice, Likert scale) were not mentioned.
3) The use of an online survey platform is practical but might exclude students with limited internet access or digital literacy. This potential limitation should be acknowledged.
4) The survey was conducted over a very short period (three days). Was this duration sufficient to ensure adequate participation? Details on response rates and efforts to maximize participation should be included.
5) Although participant anonymity is mentioned, there is no explanation of how data confidentiality was maintained or secured during the online survey process.

**RESULT**

The Results section provides a detailed presentation of findings, including descriptive statistics, group comparisons, and interpretations of data. However, there are several strengths and areas for improvement in clarity:

1) Page 7, line 2: “P < 0” is incomplete and needs to be corrected to “p < 0.001.”
2) Table 1 is hard to follow due to inconsistent formatting. The alignment of percentages and group totals should be clearer, and column labels could be more descriptive (e.g., “Exposure mode” instead of “Subtotal”).
3) Some findings are described as significant, but their practical implications are not discussed. For example:
   a. While students from endemic areas demonstrated greater knowledge of symptoms, the impact on prevention behaviors is not addressed.
   b. A lack of significant differences (e.g., exposure patterns between endemic and nonendemic students) is mentioned without further exploration of why this might be the case

**DISCUSSION**

The discussion section contextualizes the findings within the broader framework of schistosomiasis control and highlights areas for improvement in health education strategies for university students. Here are few areas for improvement:

1) While findings are summarized, the discussion lacks a deeper exploration of their implications. For example:
   a. Why is knowledge of schistosomiasis so low among university students despite health education efforts in primary and secondary schools?
   b. How might the identified preferences for multimedia education impact the scalability and sustainability of interventions?
2) The discussion does not address the study’s limitations, such as potential biases in self-reported data, limited generalizability, or the cross-sectional nature of the study.
