# Supplementary material for: Research on health education and health promotion during the process of schistosomiasis elimination II Awareness among university students in endemic regions
Source: PLoS Negl Trop Dis. 2025 Feb 18;19(2):e0012865. doi: 10.1371/journal.pntd.0012865 (PMC11835237; doi:10.1371/journal.pntd.0012865)
Supplement: Attachment — Submitted filename: 20250103Response to Reviewers.docx [file pntd.0012865.s004.docx]

**Dear Reviewers:**

Thank you for your interest in our manuscript entitled “Research on health education and health promotion during the process of schistosomiasis elimination II A study examining the awareness of schistosomiasis among students at higher education institutions in endemic regions”. We have studies comments carefully and have made correction which we hope meet the final approval. The alterations in the text are highlighted in the paper as requested. The main corrections in the paper and the responses to the reviewer’s comments are as following:

**Topic**

Reviewer #1:

The title is overly long and slightly ambiguous. Consider simplifying it while retaining key. Here is a suggestion: "Health Education and Promotion During Schistosomiasis Elimination: Awareness Among Students in Endemic Regions".

**Response:** Thank you very much. As this is part of a series of studies on "**Research on health education and health promotion during the process of schistosomiasis elimination**", the main title cannot be changed. The main title cannot be changed, but we agree with your suggestion for the subtitle and have revised it to "***Awareness among university students in endemic regions***".

**Abstract**

Reviewer #1:

The abstract provides a comprehensive overview of the research. However, it is rather too long with lots of redundancies. Authors are advised to rephrase and summarize the abstract section. There are areas where the clarity, and structure could be improved:

1)	The objectives aspect introduces schistosomiasis and health education in China, but it lacks a clear framing of why university students are a critical focus for this study. **Briefly connecting this to their role as a potentially at-risk or influential group would enhance its relevance**.

2)	The description of the questionnaire's content is helpful but could be more concise. For example, instead of listing all topics, summarize: *"The survey assessed exposure to snail-infested water, knowledge about schistosomiasis, and preferences for health education methods."*

3)	Some statistical data (e.g., “χ²=0.620, P>0.05”) are included without sufficient context. If the finding is not significant, it might be better to emphasize the broader pattern rather than the statistical test result, which may distract a general audience. The result section can be summarized below:

*"Among 4,847 respondents (49% male, 51% female), 53.8% reported exposure to snail-infested environments near their schools, and 38.4% had direct contact with snail-infested water. Awareness of schistosomiasis was low (31.1%), with limited knowledge about transmission (30.6%) and symptoms (42.9%). Preferred education methods included mobile multimedia (72.8%), brochures (68.7%), and physical promotional items (66.6%)"*.

**Response:** We are in agreement with your suggestions and have rewritten the abstract as follows:

*"In China, health education serves as the primary method for controlling schistosomiasis and has significantly contributed to the management of schistosomiasis epidemics. In contrast, university students who live/study in schistosomiasis-endemic areas are often in the risk zone for schistosomiasis infection as part of their social practice and leisure activities. Thus, the risk of schistosomiasis transmission remains widespread and chronic. This study will conduct a survey and analyze schistosomiasis awareness among university students in colleges and universities in endemic areas. The aim is to optimize intervention strategies once transmission has been interrupted. Students from two colleges and universities in the city of Gongqingcheng on Lake Poyang were selected and asked to complete a questionnaire via the online platform Questionnaire Star (https://www.wjx.cn/). The survey assessed exposure to snail-infested water, knowledge about schistosomiasis, and preferences for health education methods. The survey took place from April 2 to April 4, 2024. The survey results show that of the 4,847 respondents (49% male, 51% female), 53.8% reported exposure to snail-infested environments near their schools, and 38.4% had direct contact with snail-infested water. Awareness of schistosomiasis was low (31.1%), with limited knowledge about transmission (30.6%) and symptoms (42.9%). Preferred education methods included mobile multimedia (72.8%), brochures (68.7%), and physical promotional items (66.6%). In general, the overall awareness of schistosomiasis among university students remains low. There is a need to enhance health education in schools to improve disease prevention awareness within this population."*

**Introduction**

Reviewer #1:

The introduction effectively highlights schistosomiasis as a significant public health concern, contextualizing its global and regional impact. By referencing government initiatives and WHO strategies, it establishes credibility while emphasizing China's progress, including the 2023–2030 Action Programme for Schistosomiasis Elimination. The identified gaps in health education for mobile populations emphasize the study's importance and provide a clear rationale for its focus. However, there is need for some improvements:

1)	While the gaps are identified, the justification for focusing specifically on university students could be stronger. Briefly linking this to their unique vulnerability or potential role as change agents in endemic areas would add value.

**Response:** This is a very good suggestion, and we have added the following to the introduction：

*"University students are more vulnerable to mental health crises when faced with issues such as academic stress, relationships and punishment, and this vulnerability means they need more support and guidance to cope with the challenges, especially in schistosomiasis-endemic areas where they may face additional health and environmental burdens. Health education for university students is an important part of improving health for all. As future leaders and professionals, the health awareness and behaviours of university students will have a long-term impact on society. In schistosomiasis-endemic areas, university students can raise community awareness of schistosomiasis and promote preventive measures through health education activities."*

2)	Paragraph 3: Kindly rephrase this sentence for more clarity "*China has gained much experience in health education and health promotion in the process of schistosomiasis prevention and treatment, which has provided some technical support for the elimination of schistosomiasis*"

**Response:** OK, revised as follows:

*"Throughout its efforts to control and prevent schistosomiasis, China has gained considerable expertise in health education and promotion, providing valuable technical assistance in the effort to eradicate the disease."*

**Methods**

Reviewer #1:

1)	The choice of a non-probability sample survey is mentioned but not justified. Why this method was selected over a probability sampling method should be explained, as it affects the generalizability of the results.

**Response:** The rationale for the choice of the 'non-probability sample survey' has been further explained in the methodology. These are described below:

*"Since the target population of this survey is university students, and considering the unpleasant and troublesome phenomenon of respondents in the survey process, the method of using voluntary respondents as the survey sample, i.e., the non-probability sample survey method, is adopted."*

2)	While the questionnaire topics were briefly described, more detail is needed about how it was developed and validated. Also, the format and scope of the questions (e.g., multiple choice, Likert scale) were not mentioned.

**Response:** The way in which the questionnaire has been developed is described in the methodology, i.e. *"The questionnaire was self-designed and developed in consultation with several schistosomiasis control and health education experts."* The following additions were made to the format and scope of the questionnaire:

*"It consists of 4 multiple-choice questions, 1 fill-in-the-blank question and 13 single-choice questions."*

We also accepted your suggestion to validate the questionnaire and the results of the validation showed that the questionnaire was designed with good reliability and validity of 0.819 and 0.857 respectively (added to the methodology). The results of the analysis are presented below:

We have now translated the questionnaire into English and uploaded it to the submission system as an attachment for your reference.

3)	The use of an online survey platform is practical but might exclude students with limited internet access or digital literacy. This potential limitation should be acknowledged.

**Response:** Thank you for your valuable feedback on our research methodology. We understand your concern that online survey platforms may exclude certain groups. However, given that our respondents were students, we believe this concern had limited impact in this study. Below are our thoughts:

**Internet access for students:** In today's educational environment, university students generally have better access to the Internet. Most universities and colleges provide campus networks, and student residences, libraries and study centres usually have Wi-Fi coverage. In addition, many students own personal computers or smartphones that provide easy access to the Internet.

**Digital literacy:** University students tend to be highly digitally literate. They regularly use online resources and tools in their studies and are relatively proficient in completing tasks such as online questionnaires. Therefore, we believe that respondents were able to participate in the online survey without any barriers.

**Technological adaptability:** As the university student population is more adaptable to new technologies, they are more likely to have the basic skills needed to complete online surveys. In addition, colleges and universities usually provide the necessary technical support to help students solve problems they may encounter when using online platforms.

Based on these considerations, we believe that an online survey platform is appropriate for the purpose of our study and can reach the vast majority of college student respondents.

4)	The survey was conducted over a very short period (three days). Was this duration sufficient to ensure adequate participation? Details on response rates and efforts to maximize participation should be included.

**Response:** We are very sorry that we did not describe the sample size calculation in our article, so we have included the sample size calculation in our methodology. According to our calculations, the minimum sample size of 1062 for this research study would be sufficient for the study. Due to the strong diffusion of the network, coupled with the high enthusiasm of the students to participate, this survey can be completed in a very short time. The sample size calculation was added as follows:

*"The formula for calculating the sample size for the required survey is* $n=\frac{Z^{2}\cdot p(1-p)}{E^{2}}$*, where Z is the 95% confidence level (1.96), p is the estimated total proportion (since it is unknown, it is conservatively taken as 0.5, which ensures that the variance is maximised at this point) and E is the maximum permitted error (3%). For this reason, the minimum sample size required for this survey is approximately 1062. "*

5)	Although participant anonymity is mentioned, there is no explanation of how data confidentiality was maintained or secured during the online survey process.

**Response:** Thank you for your concern about the confidentiality of the data in our study. Your comments are very pertinent and we share your recognition of the importance of maintaining data confidentiality during the online survey process and have given this much thought. For this reason, we have included additional descriptions in our methodology regarding the maintenance of data confidentiality. These are described below:

*"Data anonymisation and pseudo-anonymisation techniques were used to protect the privacy of participants. This involves removing all personal identifiers from the data and replacing identifying details with artificial identifiers, which effectively reduces the risk of revealing participants' identities. At the same time, we ensure that data is stored using encrypted methods, whether on local servers or in the cloud. We also regularly back up stored data in case a cyber event results in data loss. "*

Reviewer #2:

1. There is no clear criterion for choosing the 2 out of the 14 institutions or colleges

**Response:** Thank you very much for your question about the criteria of the schools we chose, this is a big mistake in our description, because we did not conduct this survey only in these 2 universities, but at the same time as the students of these 2 universities took the questionnaire, it was distributed by these respondents to the students of the other 12 universities located in Gongqingcheng through the WeChat push function, like a neural net-like diffusion. Unfortunately, the survey was anonymous and we don't know how many schools were included. Therefore, we have revised it as follows:

*"After the respondents in the two universities completed the questionnaire, the link to the questionnaire was pushed to the students in the other 12 universities via WeChat (social software)."*

2. Although it was mentioned that nonprobability sampling was used, the paper did not describe how participants were selected and recruited, and how they were given access to the questionnaire. This information is necessary to ensure that participation was indeed voluntary and that the manner of recruitment did not include any undue influence.

**Response:** We have provided additional clarification, which is set out below:

*"The students to be interviewed were approached by the counsellors of the institutions and completed the questionnaire on the condition that their participation was voluntary (they could refuse and would not be subjected to any unfair treatment)."*

3. RESULTS: "A total of 4,847 university students were interviewed..." were there interviews done or all participants answered the online questionnaire (self-administered)?

**Response:** Revised to read "responded the online questionnaire".

4. present the average or median age instead of using proportions since the age grouping used included almost all the study participants

**Response:** It is very unfortunate that we did not include the specific age in our questionnaire design, but rather designed the age range to allow for a more direct choice by the respondent to avoid the mistake of entering the age directly. We have now translated the questionnaire into English and uploaded it to the submission system as an attachment for your reference.

5. It is unclear why the authors are comparing the exposure to snail water of students from endemic and non-endemic areas. Throughout the paper, the authors focused on comparing water contact exposure and knowledge of students from endemic and non-endemic areas prior to arriving in the university, when the aim tries to assess overall exposure and knowledge. If the significance of the study lies in determining whether there is a need to implement health education campaigns in universities in the area, there is really no value to testing the hypothesis whether students from endemic and non-endemic areas have differences in exposures and knowledge, since interventions will not make a distinction between these two types of students when implemented.

**Response:** When we considered comparing respondents from schistosomiasis endemic and non-endemic areas, in addition to determining the need for schistosomiasis health education activities for university students, we also needed to assess the sustainability of knowledge about schistosomiasis control among university students from schistosomiasis endemic areas, but it is clear that the results were unsatisfactory and how this unsatisfactory phenomenon occurred. We have analysed this in the discussion section.

6. It was understandable that the questions on exposures to snail water or areas where there may be snail populations were only administered to those who went to areas where snails may be present. However, those who did not go to such areas should have been asked to answer the knowledge questions too. Excluding them from this part of the survey may constitute selection bias, and may introduce errors in the estimates of level of knowledge of the study population. Those students who refrained from going to areas where there may be snail populations may have done so because they have knowledge of schistosomiasis transmission.

**Response:** This is indeed a point that you raise and one that we had not considered, thank you very much. Unfortunately, given that the original design of our questionnaire excluded this population and that this was an anonymous survey, we are unable to add to it retrospectively. However, we will compensate for the shortcomings of this survey by continuing to focus on this population in future studies of the intervention. In addition, in another survey in our series of studies of tourists travelling to at-risk areas for recreation, we found that the level of knowledge about schistosomiasis control did not seem to influence their decision to travel to at-risk areas.

7. what test was done to compare the recognition of mobile multimedia against other health education and publicity?

**Response:** This is a cross-sectional study to find out the "**acceptability of schistosomiasis health education and promotion methods**" among university students. The next step in our research will be to find out which health education methods are more effective for university students.

**Results**

Reviewer #1:

1)	Page 7, line 2: “P < 0” is incomplete and needs to be corrected to “p < 0.001.”

**Response:** Revised to p<0.01.

2)	Table 1 is hard to follow due to inconsistent formatting. The alignment of percentages and group totals should be clearer, and column labels could be more descriptive (e.g., “Exposure mode” instead of “Subtotal”).

**Response:** The table has been converted to a number format to make it easier to read.

**Fig. 2 Percentage of different types of exposure to snail-infested water**

3)	Some findings are described as significant, but their practical implications are not discussed. For example:

a.	While students from endemic areas demonstrated greater knowledge of symptoms, the impact on prevention behaviors is not addressed.

**Response:** The question you raise is very valid and we agree with it. For this reason, we have added to the discussion section to address this issue. The details are as follows:

*"Schistosomiasis is a serious public health problem transmitted by contact with fresh water containing larvae, causing symptoms such as anaemia, diarrhoea and abdominal pain and, in severe cases, cirrhosis of the liver. Knowledge of the symptoms is essential for early detection and treatment. The survey showed that knowledge of symptoms among university students in endemic areas (50.17%) was significantly higher than in non-endemic areas (38.80%), but knowledge of symptoms alone is not enough to effectively prevent transmission. Prevention of schistosomiasis requires a combination of individual knowledge and community and policy support. China has undertaken a large number of health education efforts on prevention and treatment, including the production of guidebooks and evaluation programmes. These efforts have shown that education and public awareness can be effective in changing behaviour and reducing transmission. Therefore, in endemic areas, in addition to raising awareness of symptoms, there is a greater need to promote preventive behaviours through health education and community participation to more effectively control and prevent schistosomiasis transmission."*

b.	A lack of significant differences (e.g., exposure patterns between endemic and nonendemic students) is mentioned without further exploration of why this might be the case.

**Response:** Yes, we have further analysed the possible reasons for this phenomenon in the discussion section. The specific additions are as follows:

*"Although the proportion of students in non-endemic areas exposed to snail water from digging wild vegetables was significantly higher than that in endemic areas (P<0.001), overall there was no significant difference in the proportion of exposure between students in endemic and non-endemic areas. Possible reasons include: 1) insufficient health education coverage, schistosomiasis education in endemic areas did not comprehensively cover the target population, resulting in insufficient awareness of the risk of schistosomiasis; 2) similar behavioural habits, university students in endemic and non-endemic areas faced the same risk of schistosomiasis exposure during activities such as swimming and playing in water; 3) Similar environmental factors, natural water bodies such as lakes and rivers were present in endemic and non-endemic areas, which may lead to unintentional exposure to infected water; 4) Inadequate community participation, if community participation and implementation of schistosomiasis control measures are weak, it may lead to no significant difference in the exposure behaviour of university students in the two districts. It is suggested that university students are at risk of schistosomiasis infection during outdoor activities."*

Reviewer #2:

Is there a way to ascertain that the students who went to the high risk areas were really exposed to snails? Because the way some parts of the discussions were written seem to indicate certainty of exposure (e.g. A total of 25.64% of the students were exposed to snails during the susceptible season from April--October, whereas 25.95% were exposed to snails throughout the year (irregular)). A visit to the area which are known to be habitats of the snail may be described as possible exposure instead of labeling them as exposed.

**Response:** We apologise for any misunderstanding you may have had due to our language, we really meant 'snail water' not 'snails'. We have revised it.

There was no clear description of how the visit to the snail area was asked: was it a visit in the past year? past 2 years? did the authors consider and collect data for multiple visits? Is there data on the number of students with multiple visits?

**Response:** Sorry, we did not consider the frequency of university students travelling to areas with snails, it is a complex project and not what we needed for this survey. A simple retrospective survey of frequency would be highly biased and a prospective study with a cohort is needed to address this issue.

Reviewer #3: data analysis and results are well presented. The study aimed to optimize interventions and strategies once transmission has been interrupted. I was of the opinion that rather than just ask participants if they would accept schistosomiasis health education and publicity; the authors would outline the health education and health promotion strategies as well as put them to test to see which components of the interventions are effective and/or which need to be strengthened for maximum impact. this would allow them the opportunity to then analyze data to identify effective elements and refine the different health education and health promotion components accordingly.

**Response:** This is a very good proposal. The current survey is a cross-sectional study to investigate the acceptability of "**acceptability of schistosomiasis health education and promotion methods**" among university students. The next step in our study will be to determine which health education methods are more effective for university students.

the finding that "knowledge of schistosomiasis among the university students revealed that only 31.08% of them knew about the disease, and 30.58%, 49.20%, 30.33% and 42.91% of these university students had no knowledge of "how to contract schistosomiasis", "how to identify risk areas for infection", "which organisms transmit schistosomiasis" and "what are the symptoms of schistosomiasis", was interesting. Since schistosomiasis health education as per China Action Programme to accelerate the achievement of the goal of eliminating schistosomiasis (2023--2030) and the Regulations on the Prevention and Treatment of Schistosomiasis.

Why has health education on the disease leave out university students?

**Response:** In response to the question, "Why has health education on the disease leave out university students?", it is precisely because of this phenomenon, which we have seen in the fight against schistosomiasis but which we do not have the data to illustrate, that these surveys were carried out to remind decision-makers not to ignore them.

Similarly, could there be recall bias on the part of participants?

**Response:** Students who are able to enter university still have a relatively strong memory and although there may be a recall bias, this phenomenon should be an isolated case in the overall population and will not affect the final conclusions.

The study also does not state level of students who participated in the study; e.g. where they finalists or beginners?

**Response:** Thank you very much for looking into this, it is something we had not considered. As we answered in the previous question, their memory is very strong and will not affect the final result.

could there have been overlaps in the questions and/or responses of participants?

**Response:** Each person can only take part in one survey, which is the function of the online platform 'Questionnaire Star' (https://www.wjx.cn/).

**Conclusions**

Reviewer #1:

The discussion section contextualizes the findings within the broader framework of schistosomiasis control and highlights areas for improvement in health education strategies for university students. Here are few areas for improvement:

1)	While findings are summarized, the discussion lacks a deeper exploration of their implications. For example:

a.	Why is knowledge of schistosomiasis so low among university students despite health education efforts in primary and secondary schools?

**Response:** In response to the question, "Why has health education on the disease leave out university students?", it is precisely because of this phenomenon, which we have seen in the fight against schistosomiasis but which we do not have the data to illustrate, that these surveys were carried out to remind decision-makers not to ignore them.

b.	How might the identified preferences for multimedia education impact the scalability and sustainability of interventions?

**Response:** This is a very good proposal. The current survey is a cross-sectional study to investigate the acceptability of "**acceptability of schistosomiasis health education and promotion methods**" among university students. The next step in our study will be to determine which health education methods are more effective for university students.

2)	The discussion does not address the study’s limitations, such as potential biases in self-reported data, limited generalizability, or the cross-sectional nature of the study.

**Response:** We have made the following additions to the discussion section:

*"This study has several limitations. Firstly, the sample was exclusively sourced from a single province in China where schistosomiasis is endemic. Secondly, the brief duration of the survey and the anonymity of participants precluded the possibility of conducting repeat testing. Additionally, we did not evaluate the knowledge of schistosomiasis prevention among university students who did not visit regions with snail populations. Nonetheless, our findings are currently preliminary, indicating that further research is essential to validate these results."*

Reviewer #2: No description of the limitations of the study and how these were addressed.

**Response:** We have made the following additions to the discussion section:

*"This study has several limitations. Firstly, the sample was exclusively sourced from a single province in China where schistosomiasis is endemic. Secondly, the brief duration of the survey and the anonymity of participants precluded the possibility of conducting repeat testing. Additionally, we did not evaluate the knowledge of schistosomiasis prevention among university students who did not visit regions with snail populations. Nonetheless, our findings are currently preliminary, indicating that further research is essential to validate these results."*

Reviewer #3: The authors make suggestions of the different communication approaches to be adopted for health education for different population groups but are not explicit about public health relevance. Limitations not clearly articulated.

**Response:** We have made the following additions to the discussion section:

***Supplement 1****："Schistosomiasis is a serious public health problem transmitted by contact with fresh water containing larvae, causing symptoms such as anaemia, diarrhoea and abdominal pain and, in severe cases, cirrhosis of the liver. Knowledge of the symptoms is essential for early detection and treatment. The survey showed that knowledge of symptoms among university students in endemic areas (50.17%) was significantly higher than in non-endemic areas (38.80%), but knowledge of symptoms alone is not enough to effectively prevent transmission. Prevention of schistosomiasis requires a combination of individual knowledge and community and policy support. China has undertaken a large number of health education efforts on prevention and treatment, including the production of guidebooks and evaluation programmes. These efforts have shown that education and public awareness can be effective in changing behaviour and reducing transmission. Therefore, in endemic areas, in addition to raising awareness of symptoms, there is a greater need to promote preventive behaviours through health education and community participation to more effectively control and prevent schistosomiasis transmission."*

***Supplement 2****："Although the proportion of students in non-endemic areas exposed to snail water from digging wild vegetables was significantly higher than that in endemic areas (P<0.001), overall there was no significant difference in the proportion of exposure between students in endemic and non-endemic areas. Possible reasons include: 1) insufficient health education coverage, schistosomiasis education in endemic areas did not comprehensively cover the target population, resulting in insufficient awareness of the risk of schistosomiasis; 2) similar behavioural habits, university students in endemic and non-endemic areas faced the same risk of schistosomiasis exposure during activities such as swimming and playing in water; 3) Similar environmental factors, natural water bodies such as lakes and rivers were present in endemic and non-endemic areas, which may lead to unintentional exposure to infected water; 4) Inadequate community participation, if community participation and implementation of schistosomiasis control measures are weak, it may lead to no significant difference in the exposure behaviour of university students in the two districts. It is suggested that university students are at risk of schistosomiasis infection during outdoor activities."*

***Limitations：****This study has several limitations. Firstly, the sample was exclusively sourced from a single province in China where schistosomiasis is endemic. Secondly, the brief duration of the survey and the anonymity of participants precluded the possibility of conducting repeat testing. Additionally, we did not evaluate the knowledge of schistosomiasis prevention among university students who did not visit regions with snail populations. Nonetheless, our findings are currently preliminary, indicating that further research is essential to validate these results.*

**Editorial and Data Presentation Modifications?**

Reviewer #2: Table 1: what is under the subtotal column? the number and % exposed? then it should be labeled as that, because this column does not show the subtotal of the columns on the left.

**Response:** The table has been converted to a number format to make it easier to read.

**Fig. 2 Percentage of different types of exposure to snail-infested water**

Figure 1: The figure, based on the write up, is intended to show the proportion of students who visited the area on months when there is high susceptibility to exposure and infection. Maybe label the April to October as the susceptible months and the others as non-susceptible (can Jan-Mar and Nov-Dec be combined under this?). Also, please edit the title if the labels will be changed.

**Response:** Thank you for your suggestion, after research and discussion by our project team we have decided not to revise it. Because it may be more intuitive for readers to understand.

Reviewer #3: Perhaps the authors should be more explicit about the specific components of health education and health promotion strategies and interventions that were optimized in this study and describe the process. Just asking participants to state their willingness to accept health education and publicity, through mobile multimedia, brochures etc while it is important doesn't seem adequate if the goal is to optimize these strategies.

**Response:** This is a very good proposal. The current survey is a cross-sectional study to investigate the acceptability of "**acceptability of schistosomiasis health education and promotion methods**" among university students. The next step in our study will be to determine which health education methods are more effective for university students.

**Summary and General Comments**

Reviewer #2:

The study has elucidated the significance of doing the survey in the specific study population. However, the direction of the analysis and discussion do not seem to address this significance very well. A comparison of the knowledge of those who visited and did not visit the high risk area may be more relevant in terms of planning for interventions. This can help determine whether people risk going to areas with snail populations because they have less knowledge on schistosomiasis as compared to those who avoided visiting those sites. If this is the case, then maybe an intervention to inform the students not only of schistosomiasis but of the risk that is present in the areas may be helpful.

**Response:** This is indeed a point that you raise and one that we had not considered, thank you very much. Unfortunately, given that the original design of our questionnaire excluded this population and that this was an anonymous survey, we are unable to add to it retrospectively. However, we will compensate for the shortcomings of this survey by continuing to focus on this population in future studies of the intervention. In addition, in another survey in our series of studies of tourists travelling to at-risk areas for recreation, we found that the level of knowledge about schistosomiasis control did not seem to influence their decision to travel to at-risk areas. So the results of this survey still tell us something.

Reviewer #3:

Overall, there is scientific validity. The authors acknowledge the contagious and debilitating nature of schistosomiasis globally and specifically in China. the article is well written and presented in a scholarly manner. However, the authors must align their objective on optimization of health education strategies to their findings and conclusions. I think that the authors should also consider health education and health promotion for those communities in endemic areas for sustainable control and prevention of schistosomiasis.

**Response:** Thank you very much. Our survey is part of the series "Research on health education and health promotion during the process of schistosomiasis elimination". Further prospective cohort studies are needed to investigate the optimisation of health education strategies in different populations.
