# Supplementary material for: Research on health education and health promotion during the process of schistosomiasis elimination II Awareness among university students in endemic regions
Source: PLoS Negl Trop Dis. 2025 Feb 18;19(2):e0012865. doi: 10.1371/journal.pntd.0012865 (PMC11835237; doi:10.1371/journal.pntd.0012865)
Supplement: Attachment — Submitted filename: 20250120Response to Reviewers.docx [file pntd.0012865.s005.docx]

**Dear Editors & Reviewers:**

We thank the editors and reviewers for the kind consideration and constructive comments on our manuscript. We have carefully revised the manuscript. The alterations in the text are highlighted in the paper as requested. We have addressed your concerns in a point-by-point manner below, and hope that you will find the added information suitable and sufficient for publication.

**Questionnaire**

Some questions seem to be in the future tense including Q6 which asks “Which of the following activities will you engage in when you go to the Poyang Lake area?” This may be an accident of translation but needs to be changed if it was not asking about future visits to the lake.

Q11 seems to have two correct answers, Oncomelania and snails. How was this handled?

Q14 what is “blood-prevention”? This may be a translation issue. Maybe just use “prevention”?

**Response:** Thank you for pointing out the deficiencies in the translation of our questionnaire, we have reorganised and revised the entire questionnaire. The details are as follows:

*Q1:* *"Will you visit the surrounding areas of Poyang Lake, such as Nanhu, Sizhuang, Wanjia Lake, Sujiadang, etc., and do activities there?" → "Have you visited the surrounding areas of Poyang Lake, such as Nanhu, Sizhuang, Wanjia Lake, Sujiadang, etc., and done activities there?"*

*Q6: "Which of the following activities will you engage in when you go to the Poyang Lake area?" → "**Which of the following activities have you done since arriving in the Poyang Lake District?"*

*Q11: "D. Snail" → "D. Semisulcospira cancellata"*

*Q14: " Have you seen a blood-prevention warning sign or heard a voice prompt on the side of the road, or received a blood-prevention publicity leaflet when participating in activities in this area?" → "**Have you ever seen a roadside sign or audio message about schistosomiasis control or received a leaflet about schistosomiasis control when entering this area? "*

**General**

Much of the manuscript is written in the future tense. Manuscripts should always be in the past tense since they describe work that was conducted in the past.

**Response:** OK. We have re-touched the entire text.

**Abstract**

Make it clear that the knowledge questions were only answered by students that visited the lake area.

**Response:** OK. Your suggested changes are spot on and we have incorporated them into the manuscript as you suggested. Those marked in red are additions, as follows:

*"The survey assessed exposure to snail-infested water, knowledge about schistosomiasis, and preferences for health education methods among students who had traveled to snail breeding areas."*

One of the key objectives and outcomes reported in the manuscript is comparing students from endemic and non-endemic areas and yet there is no mention of this in the abstract nor the key significant differences found and their implication for policy.

**Response:** We revise and add to this section as follows:

***Revised:*** *"This study will conduct a survey and analyze schistosomiasis awareness among university students in colleges and universities in endemic areas." → "* *This study will conducted a survey and analyzed schistosomiasis awareness among university students in colleges and universities in endemic areas."*

***Supplement 1:*** *"* Of these, with those who dug for wild vegetables exposed significantly more nonendemic than endemic students to snail water (13.93% vs. 8.42%, χ^2^ = 16.681, P = 0.000).*"*

***Supplement 2:*** *"* Of these, students from endemic areas were significantly more aware of transmission and symptoms than those from nonendemic areas (36.52% vs. 27.22%, χ^2^= 7.623, P = 0.006 & 50.17% vs. 38.80%, χ^2^ = 9.872, P = 0.002).*"*

**Methods**

Thank you for adding a description of the sample size. Please add a statement about how long the survey instrument was available online. What was the trigger for it to be withdrawn/blocked? The sample size was calculated as 1062 and yet you report responses from 4847 students.

**Response:** Thank you for pointing this out. At the end of *"*Methods*"* --> *"*Research Methodology*"*, we mentioned the start and end dates of the survey, but did not give the reason for blocked the survey. We have made the following changes and additions.

*"The survey was conducted from 2 to 4 April 2024." → "The survey on the web platform started on 2 April 2024. By 4 April, the sample size of the survey had fully met our survey needs, so we blocked the survey instrument on the web platform."*

If following STROBE ideally you would include a flow chart of the student numbers at each stage: # approached by counsellors, # responded, # visited lake and responded to knowledge questions. Preferably split by endemicity of origin since that is your main comparator.

**Response:** That's a very good suggestion, thank you! We have included it in the main text. The details are as follows:

*"Fig. 2 showd the change in the number of respondents for the different contents of the questionnaire.*

***Fig. 2 Changes in the number of respondents****"*

Line 164: this still refers to interviews. Did the counsellors interview the students or were they given a link to access the online questionnaire? This needs to be clear.

**Response:** It has been revised and is shown below before and after the revision:

*"The students to be interviewed were approached by the counsellors of the institutions and completed the questionnaire on the condition that their participation was voluntary (they could refuse and would not be subjected to any unfair treatment)." → "The students interviewed were contacted by the counsellors of the institutions and procided with a link to the questionnaire of the platform, which they filled in on the condition of voluntary participation(they could refuse and would not be subjected to any unfair treatment)."*

In the response letter there is an extensive justification of using the online platform but this does not appear in the manuscript. I recommend adding one sentence to justify the platform.

**Response:** We have made the addition, thank you for your suggestion.

*"The "Questionnaire Star"(https://www.wjx.cn/) platform was able to reach respondents quickly due to its efficient data collection and support for multiple distribution channels such as QR code, SMS, email, website embedding, etc."*

Page 7 line 168. Explain the process of selection of the first two universities.

**Response:** OK. These two universities were randomly selected with the aim of using respondents from these two universities to spread the link to the questionnaire to the other 12 universities. We have provided an additional explanation of the methodology for selecting the two universities in the manuscript.

*"At the beginning, we randomly selected two universities to send the link to. "*

Page 7 Line 173: many of your questions were multiple choice but in some only a single answer was allowed and in four of them multiple options could be selected. Describe this correctly.

**Response:** OK. We have made some revisions to it.

*"It consists of 4 multiple-choice questions, 1 fill-in-the-blank question and 13 single-choice questions." → "It consists of 4 multiple options could questions, 1 fill-in-the-blank question and 13 single options could questions."*

line 174. Add to your new sentence “(the full questionnaire is available in Supporting Information S1)”.

**Response:** OK. We have made additions.

Line 175: there is no question that asks the mode of travel to the lake. Change this sentence.

**Response:** OK. We have made some revisions to it.

*"whether or not they had travelled to a snail environment around the school"*

Page 7. You have a section with definitions. You have been asked to add to this some definitions for other phrases that you use e.g. “snail water”, “field practices”.

**Response:** OK. We revised "Area type definitions" to "Definition of phrases" and added the definitions of "snail water", "field practice".

*"Snail water, is the natural habitat of the snail, such as the lake where the snail is found.*

*Field practices, refers to activities that are implemented or carried out in a real or practical setting, as opposed to theory."*

**Results**

Where p-values for the Chi2 tests are quoted they should be the actual p value, not just p<0.01 etc.

**Response:** OK. We have replaced all ranges of *P* values in the manuscript with actual values.

Page 8 line 213. There is nowhere in the questionnaire where you ask about duration of exposure. This heading and related text and Fig 3 caption should be changed. You only have the percentage of students who reported visiting the lake in specific time periods (groups of months) which you pre-set.

You could change the subtitle to: Type and period of exposure to snail water

Change Fig 3 caption to: Time of year students from different regions visited snail environments

**Response:** Your suggested changes are spot on and we have incorporated them into the manuscript as you suggested.

Line 216-218: the percentage of university students exposed to snail water through field practices was greater in endemic areas than in nonendemic areas.

You did not assess this in non-endemic areas. Please be careful with your wording. This should be changed to read “the percentage of university students exposed to snail water through field practices was greater for students from endemic areas than those from nonendemic areas.”

**Response:** OK. Your suggested changes are spot on and we have incorporated them into the manuscript as you suggested.

Fig 2 & 3 Adding an * to the figure above the bars where p<0.05 comparing endemic & nonendemic students would help the reader.

**Response:** OK. We have added '*' to Figure 2. However, in Figure 3 there is no significance between any of them.

Line 228. Please add a few words to explain why April-October is considered the “susceptible season”. Is this the rainy season when snail populations are highest?

**Response:** OK. In the "Definition of phrases" we have defined "susceptible season".

*"Susceptible season, is a period of the year when the risk of transmission of schistosomiasis increases significantly due to a combination of the natural environment and human activities. For schistosomiasis, the susceptible season is concentrated between April and October each year."*

Knowledge of schistosomiasis

The authors were asked to rewrite the results description to make it clearer which they have not done. They were also asked to explain Fig 4 better, which has not been done.

**Response:** OK. Yes, we have re-described these issues as you have listed below.

Line 244: The results of the schistosomiasis control knowledge test of 811 university students who were aware of schistosomiasis revealed that the knowledge rate of "how to contract schistosomiasis" was 30.58%, of which university students from endemic areas accounted for 36.52% of the population, which was significantly greater than that of 27.22% of the population from nonendemic areas (χ2=7.623, P < 0.01).

This is a very long sentence and should be shortened. In addition, the word “control” should be deleted since the sentences refer to knowledge of transmission, not control.

Better wording would be: “Of the 811 students who were aware of schistosomiasis only 30.58% correctly identified the mode of transmission, and significantly more students from endemic areas were correct (36.5% v. 27.22%, p= 0.00x.)”

**Response:** OK. Your suggested changes are spot on and we have incorporated them into the manuscript as you suggested.

It is really not clear what the other sentences in this paragraph are stating. Which question do they refer to and was there any difference between students from endemic and non-endemic areas.

e.g. what does this statistic refer to? which was significantly higher than that of 38.80% of the population from nonendemic areas (χ2 = 9.872, P < 0.01

**Response:** We are sorry that our limited language skills did not allow you to better understand the results described, but we have revised this paragraph to make the description clearer. The full description is as follows:

*Of the 811 students who were aware of schistosomiasis only 30.58% correctly identified the mode of transmission, and significantly more students from endemic areas were correct (36.52% vs. 27.22%, χ^2^=7.623, P = 0.006). 49.20% of students were able to identify that the area they were travelling to was a schistosomiasis transmission area and only 30.33% were able to correctly identify the intermediate host of schistosomiasis transmission, there was no significant difference in the percentage of students from endemic and nonendemic areas who were correct on these two knowledge items (52.22% vs. 47.49% & 30.72% vs. 30.12%, χ^2^ = 1.674 & 0.032, P = 0.196 & 0.858). The percentage of correct answers to the question "what are the symptoms of schistosomiasis" was 42.91%, with 50.17% of students in endemic areas gettingit right, which was significantly higher than the 38.80% of students in nonendemic areas (χ^2^ = 9.872, P = 0.002) (Fig. 5).*

Line 254: Since the reader is unlikely to know the correct answer to which facility treatment should be sought from, it is important to state that.

**Response:** We have added the following sentence to the manuscript:

*"whereas in China CDC/Schistosomiasis Control Centre was the only institution responsible for schistosomiasis control"*

Fig 4 caption or labelling should make it more clear which question in the questionnaire is presented. Why not use the same question number? Make it clear what is presented is the % of students who gave the correct answer (I assume).

**Response:** OK. Your suggested changes are spot on and we have incorporated them into the manuscript as you suggested. We have revised the title of Figure 4 as follows:

*"Correctness of university students' answers on knowledge of schistosomiasis in different regions"*

I don’t think “Knowledge 2” is “how to identify risk areas for infection”, but rather “students were aware that the areas of the lake they visited were transmission sites”. It should be correctly described based on the questionnaire.

**Response:** OK. Your suggested changes are spot on and we have incorporated them into the manuscript as you suggested.

Where are the results for questions 15, 16 and 17 which refer to prevention methods used? Why are these not presented?

**Response:** As the main purpose of this paper is to analyse preferences to understand the level of schistosomiasis knowledge and methods of schistosomiasis health education among university students, questions 15, 16 and 17 are, as you say, related to prevention methods and are therefore not included in the analysis. We will include them in the next revision if you feel it is very necessary to analyse them.

Line 269: Acceptance of schistosomiasis health education and promotion methods

I suggest this subtitle should be edited to: Mode of communication preferred by students for schistosomiasis health education

**Response:** OK. Your suggested changes are spot on and we have incorporated them into the manuscript as you suggested.

Edit the accompanying text and Fig 5 caption accordingly as they were never asked if they would accept materials, but in what mode they would prefer.

**Response:** OK. Your suggested changes are spot on and we have incorporated them into the manuscript as you suggested.

What are “physical protection promotional items”? How are they different to brochures?

**Response:** "physical protection promotional items" refers to items produced in physical form for the purpose of disseminating protection knowledge or promoting protection equipment. These promotional items are usually of a practical and educational nature and are designed to convey protection information to the public in a visual manner in order to increase the public's safety awareness and protection capability. They may include, but are not limited to, hats, fans, aprons, handbags, etc., printed with protection information.

Line 279: how is this in contrast? The previous sentence compared modes of communication. Now you compare students from different areas.

**Response:** We are sorry that you were not able to fully understand it due to a mistake in the organisation of our language, but we have re-described the result as follows:

*"Further analyses of preferences for different health education modalities among all categories of students from endemic and nonendemic areas showed no significant difference in preference rates between them (χ^2^ = 0.965 & 2.446 & 0.001 & 0.384 & 1.765, P = 0.326 & 0.118 & 0.979 & 0.536 & 0.184)"*

**Discussion**

This largely repeats the Introduction and Results. There is no discussion of the results in relation to other similar surveys on schistosomiasis knowledge and practices in China nor elsewhere.

**Response:** We have taken your advice and removed some duplicates, for example:

"The important role of health education in the prevention and treatment of schistosomiasis is emphasized in the Action Programme to accelerate the achievement of the goal of eliminating schistosomiasis (2023--2030) and the Regulations on the Prevention and Treatment of Schistosomiasis, both of which are issued by China."

"The results of the survey revealed that 53.83% of the students reported that they had visited areas with snails in the vicinity of the school. A total of 25.64% of the students were exposed to snail water during the susceptible season from April--October, whereas 25.95% were exposed to snail water throughout the year (irregular). The percentage of university students exposed to snail water in areas with snail environments was 38.37%. Although the proportion of students in non-endemic areas exposed to snail water from digging wild vegetables was significantly higher than that in endemic areas (P<0.001), "

"The knowledge of schistosomiasis among the university students revealed that only 31.08% of them knew about the disease, and 30.58%, 49.20%, 30.33% and 42.91% of these university students had no knowledge of "how to contract schistosomiasis", "how to identify risk areas for infection", "which organisms transmit schistosomiasis" and "what are the symptoms of schistosomiasis", respectively. The survey also revealed that only 34.65% of university students would visit a professional schistosomiasis control facility for testing if they suspected that they might be infected with schistosomiasis. This further indicates that there is a serious lack of knowledge about schistosomiasis prevention and treatment among university students."

These were not addressed from Reviewer #1: While findings are summarized, the discussion lacks a deeper exploration of their implications. For example:

a. Why is knowledge of schistosomiasis so low among university students despite health education efforts in primary and secondary schools?

**Response:** To address this issue, we have already discussed it in the manuscript as follows:

*"Overall, there was a serious lack of knowledge about schistosomiasis among university students.. Possible reasons included: 1) insufficient health education coverage, schistosomiasis education in endemic areas did not comprehensively cover the target population, resulting in insufficient awareness of the risk of schistosomiasis [27]; 2) similar behavioural habits, university students in endemic and nonendemic areas faced the same risk of schistosomiasis exposure during activities such as swimming and playing in water; 3) Similar environmental factors, natural water bodies such as lakes and rivers were present in endemic and nonendemic areas, which might lead to unintentional exposure to infected water; 4) Inadequate community participation, if community participation and implementation of schistosomiasis control measures were weak, it might lead to no significant difference in the exposure behaviour of university students in the two districts [28]. As a result, university students were at risk of contracting schistosomiasis while outdoors."*

b. How might the identified preferences for multimedia education impact the scalability and sustainability of interventions?

**Response:** This is a very good suggestion. However, this survey was a cross-sectional study to investigate the 'Mode of communication preferred by students for schistosomiasis health education' among university students. Our survey data could not contribute to the question ' How might the identified preferences for multimedia education impact the scalability and sustainability of interventions?'. This question will be addressed in our follow-up study.

One of the reviewers asked for more discussion of whether the higher level of knowledge of symptoms led to higher knowledge of appropriate health seeking or use of prevention methods. You added a paragraph, but it did not relate the results to each other nor to any previous publications.

**Response:** This issue has been discussed in our manuscript and literature references have been provided (Refs. 27 and 30).

**Conclusion**

Line 402: why recommend school-based prevention programs when you have shown students coming from schools in all areas of the country (? I assume) who have a low level of knowledge of schistosomiasis and prefer internet/social media-based communication? Why not just change this sentence to “internet-based education programs”?

**Response:** I am very sorry that we did not mention "recommend school-based prevention programs" in the "Conclusion" section, but "strengthen". And we also highlighted "and to actively conduct online health education to raise awareness of schistosomiasis prevention and treatment among the university student population".

**Limitations**

A short paragraph was added listing some limitations but no discussion of them and their impact on the outcomes.

**Response:** Thank you for your suggestion, we have added the ' Limitations ' section. The details are as follows, with additions in red.

*"This study had several limitations. Firstly, the sample was exclusively sourced from a single province in China where schistosomiasis was endemic, and it might not have reflect the overall picture. Secondly, we did not evaluate the knowledge of schistosomiasis prevention among university students who did not visited regions with snail populations; these students who did not visited areas with snail populations might have done so because they were aware of schistosomiasis transmission, and this may have affected the rate of correct knowledge of schistosomiasis among students. Additionally, there was a lack of information on the frequency of visits and whether the activity involved water contact (except of course swimming and washing). Nonetheless, our findings are currently preliminary, indicating that further research is essential to validate these results."*

Line 391: another limitation was that you have no way of knowing which universities respondents came from.

**Response:** We have removed this limiting factor in the manuscript.

Additionally, the lack of information on the frequency of visits and whether the activity involved water contact (except of course swimming and washing).

**Response:** OK. Your suggested changes are spot on and we have incorporated them into the manuscript as you suggested.
